# Icariin Promotes Osteogenic Differentiation in a Cell Model with NF1 Gene Knockout by Activating the cAMP/PKA/CREB Pathway

**DOI:** 10.3390/molecules28135128

**Published:** 2023-06-30

**Authors:** Meng Chen, Lianhua Lu, Dong Cheng, Jing Zhang, Xinyong Liu, Jianli Zhang, Tianliang Zhang

**Affiliations:** 1 Shandong Center for Disease Control and Prevention, Jinan 250014, China; 2 Department of Medicinal Chemistry, Key Laboratory of Chemical Biology (Ministry of Education), School of Pharmaceutical Sciences, Shandong University, Jinan 250012, China; 3 Shandong Qidu Pharmaceutical Co., Ltd., Shandong Provincial Key Laboratory of Neuroprotective Drugs, Zibo 255400, China

**Keywords:** Neurofibromatosis type 1, icariin, gene knockout, osteogenic differentiation, cAMP/PKA/CREB

## Abstract

Neurofibromatosis type 1 is a rare autosomal dominant genetic disorder, with up to 50% of patients clinically displaying skeletal defects. Currently, the pathogenesis of bone disorders in NF1 patients is unclear, and there are no effective preventive and treatment measures. In this study, we found that knockout of the NF1 gene reduced cAMP levels and osteogenic differentiation in an osteoblast model, and icariin activated the cAMP/PKA/CREB pathway to promote osteoblast differentiation of the NF1 gene knockout cell model by increasing intracellular cAMP levels. The PKA selective inhibitor H89 significantly impaired the stimulatory effect of icariin on osteogenesis in the NF1 cell model. In this study, an osteoblast model of NF1 was successfully constructed, and icariin was applied to the cell model for the first time. The results will help to elucidate the molecular mechanism of NF1 bone disease and provide new ideas for the clinical prevention and treatment of NF1 bone disease and drug development in the future.

## 1. Introduction

Neurofibromatosis type 1 (NF1), also known as von Recklinghausen disease, is an autosomal dominant disease with an incidence of 1:3000 [1]. The National Organization for Rare Disorders (NORD) defines this condition as a typical rare disorder. Clinically, NF1 is mainly characterized by peripheral nerve and brain tumors, pigmented lesions, visceral dysfunction, and distinctive skeletal lesions. Up to 50% of NF1 patients will have bone dysplasia [2]. Several skeletal manifestations are clinically associated with NF1, including osteopenia or osteoporosis, macrocephaly, short stature, scoliosis, kyphoscoliosis, chest wall and sphenoid dysplasia, and congenital tibial pseudarthrosis [3]. The most common skeletal feature is decreased bone mineral density (BMD) and an increased incidence of osteoporosis [4,5]. However, the mechanism of bone disease in NF1 patients is unclear, and there are no effective preventive and therapeutic measures. Surgery is the main treatment for skeletal disorders in NF1 patients. However, postoperative deformities may develop further, the incidence of pseudarthrosis is high, and some patients require amputation, resulting in substantial pain and economic burdens on patients [3].

Recently, gene studies have confirmed that mutation of the NF1 gene is an essential cause of NF1 [6]. The NF1 gene encodes neurofibromin, a multifunctional, cytoplasmic protein involved in several cellular signaling pathways. Its main functional area is the GAP-related domain (GRD), which has homology with the GTP-activated protein (GAP) family. This protein promotes the activated form of Ras bound to GTP to hydrolyze to the non-activated form of Ras bound to GDP, thereby acting as a negative regulator that terminates RAS-mediated signaling pathways [7]. In addition to the Ras GTPase function, neurofibromin also contains a domain rich in cysteine and serine, which may be related to regulating the activity of adenylate cyclase (AC), so the deletion of neurofibromin also leads to a dysfunctional of cAMP signaling pathway [8]. Mutations in one or two NF1 alleles result in the loss of effects of neurofibromin, leading to a dysfunctional downstream signaling pathway and ultimately to abnormal cell proliferation and tumor growth [9]. Abnormally regulated osteoblast and osteoclast activity is present in patients with NF1. Haploid dysfunction of NF1 may affect the function of bone cells, resulting in decreased bone mass and mineral content [10,11]. Data from clinical studies have demonstrated a significantly increased incidence of fractures in adult and elderly patients with NF1 compared with those not affected by the disease [12,13]. Bone biopsies from children with NF1 showed that osteoblasts in pseudo joints were less responsive to osteogenic differentiation signals than those from unaffected sites [14]. During normal intramembranous and endochondral osteogenesis in humans and animals, neurofibromin expression can be detected in osteoblasts and mature osteocytes [15]. Wu et al. demonstrated that neurofibromin plays a key role in regulating the differentiation of mesenchymal stem cells into osteoblasts in NF1+/− mice [16]. Research on a mouse model lacking the osteoblast NF1 allele revealed decreased osteoblast function and a high bone turnover phenotype characterized by increased collagen synthesis and bone formation, delayed mineralization, and increased osteoclasts mediated by RANKL [17].

Icariin, as the main active component of Epimedium, can significantly promote the calcification and maturation of osteoblasts, inhibit the bone resorption activity of osteoclasts, and induce their apoptosis [18,19]. A recent study showed that icariin could promote osteoblast gene expression and prevent osteoporosis by activating the cAMP/PKA/CREB pathway [20]. Osteopenia and osteoporosis are the most common symptoms of the skeletal system in NF1 patients [4,5,21]. The loss of neurofibromin in NF1 disease leads to abnormalities of the cAMP signaling pathway. Therefore, cAMP can be used as a key factor to link icariin to the treatment of NF1 bone diseases.

In this study, we employed a lentiviral vector carrying the CRISPR/CAS9 system to transfect C2C12 cells for targeted knockout of the NF1 gene. CRISPR/Cas9 is a technology that enables precise editing of specific sites in the genome of any species. Single or multiple gene knockouts at the cellular level can be performed using this technique. C2C12 is a subclone of a mouse myoblast cell line established by D. Yaffe and O. Saxel [22]. This line can differentiate in different directions to skeletal muscle, bone, and cartilage under different induction conditions. Therefore, the C2C12 mouse myoblast line is widely used as a model cell of undifferentiated mesenchymal cells and is often used in studying bone cell development [23,24,25]. Finally, two stable C2C12 cell lines with NF1 gene knockout were obtained, and an osteoblast differentiation cell model with NF1 gene knockout was successfully constructed. After the deletion of the NF1 gene, we found that the expression of neurofibromin was decreased, and the intracellular cAMP level was reduced compared with those of the normal control group.

Moreover, the osteogenic differentiation of the cell model was significantly impaired. Next, icariin was added during the osteogenic differentiation process of the NF1 gene knockout cell model to observe its effect. Finally, we found that icariin could promote the osteogenic differentiation of the NF1 gene knockout C2C12 cell model, and this effect was mediated by activating the intracellular cAMP/PKA/CREB signaling pathway. This research will help elucidate the molecular mechanism of NF1 skeletal disorders. This study will provide new ideas and targets for the prevention and treatment of NF1 skeletal diseases and contribute to drug research in the future. Therefore, there is important theoretical significance and application value.

## 2. Results

### 2.1. Two Lines of C2C12 Cells with NF1 Gene Knockout Were Successfully Constructed

**Figure 1:**
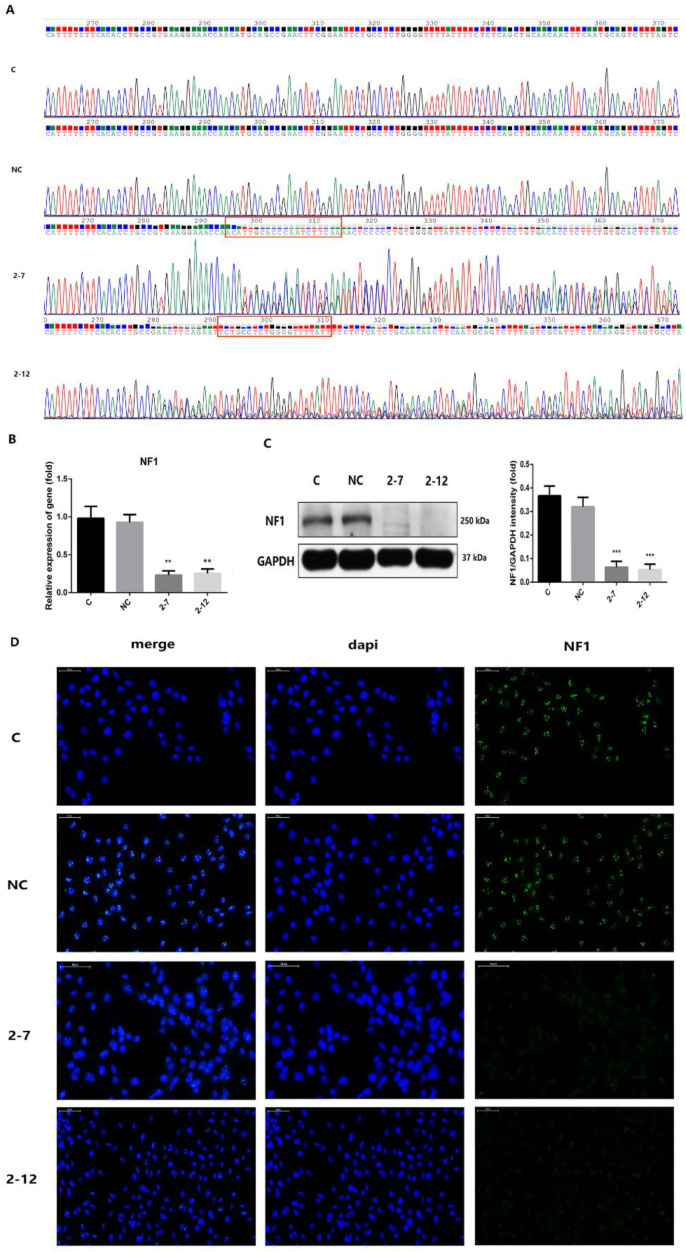
Two lines of C2C12 cells with NF1 gene knockout were constructed. (**A**) Gene sequencing of the normal control group C, negative control group NC transfected with negative lentivirus, and stable cell lines 2−7 and 2−12 transfected with positive lentivirus LV-Nf1-sgRNA. The red box shows that nesting peaks appeared at the corresponding editing position of the designed sgRNA. (**B**) The mRNA expression levels of NF1 in the four groups. (**C**) The protein levels of neurofibromin in the four groups. (**D**) The expression of neurofibromin in the four groups by immunofluorescence assays. Neurofibromin was stained green, and nuclei were stained blue (with DAPI). All data are presented as the mean ± SD (*n* = 3). ** *p* < 0.01, *** *p* < 0.001.

We used the CRISPR/Cas9 single-vector lentivirus LV-NF1-sgRNA to introduce the Cas9 protein and sgRNA sequence into C2C12 cells to knock out the NF1 gene. Through identification assays, 2−7 and 2−12 with the NF1 gene knockout were successfully selected. We divided the cells into four groups: the normal control group C, the negative control group NC transfected with negative lentivirus, and the group of stable cell lines (2−7 and 2−12) transfected with the positive lentivirus LV-NF1-sgRNA. As shown in Figure 1A, we designed and synthesized primers upstream and downstream of the sgRNA target site and performed gene sequencing on the four groups of cells. The sequencing results showed that nested peaks appeared at the position of the corresponding sgRNA in the 2−7 and 2−12 cell lines; however, group C and group NC did not show nesting peaks. The results confirmed that gene editing was carried out at the correct target after LV-NF1-sgRNA transfection. As shown in Figure 1B, the mRNA expression levels of NF1 in the 2−7 and 2−12 groups were significantly lower than those in groups C and NC. The difference was statistically significant.

Similarly, as shown in Figure 1C, the protein expression levels of neurofibromin in the four groups were evaluated by Western blotting. The results showed that the expression of neurofibromin in group C and group NC was normal. However, neurofibromin expression was undetectable in 2−7 and 2−12 cell lines. As shown in Figure 1D, immunofluorescence staining was used to detect the expression of neurofibromin in the four groups. The results showed that the expression of neurofibromin in group C and group NC was normal, and the fluorescence signal was obvious. However, neurofibromin was almost undetectable in groups 2−7 and 2−12, and no obvious fluorescence was observed. The results were consistent with those of Western blotting. Through the above series of identification, we successfully constructed two stable C2C12 cell lines with NF1 gene knockout, namely, 2−7 and 2−12. This result laid the foundation for further study of the osteogenic differentiation in cells with NF1 gene knockout.

### 2.2. The Osteogenic Differentiation and the Intracellular cAMP Level of the C2C12 Cells with NF1 Gene Knockout Were Significantly Reduced

**Figure 2:**
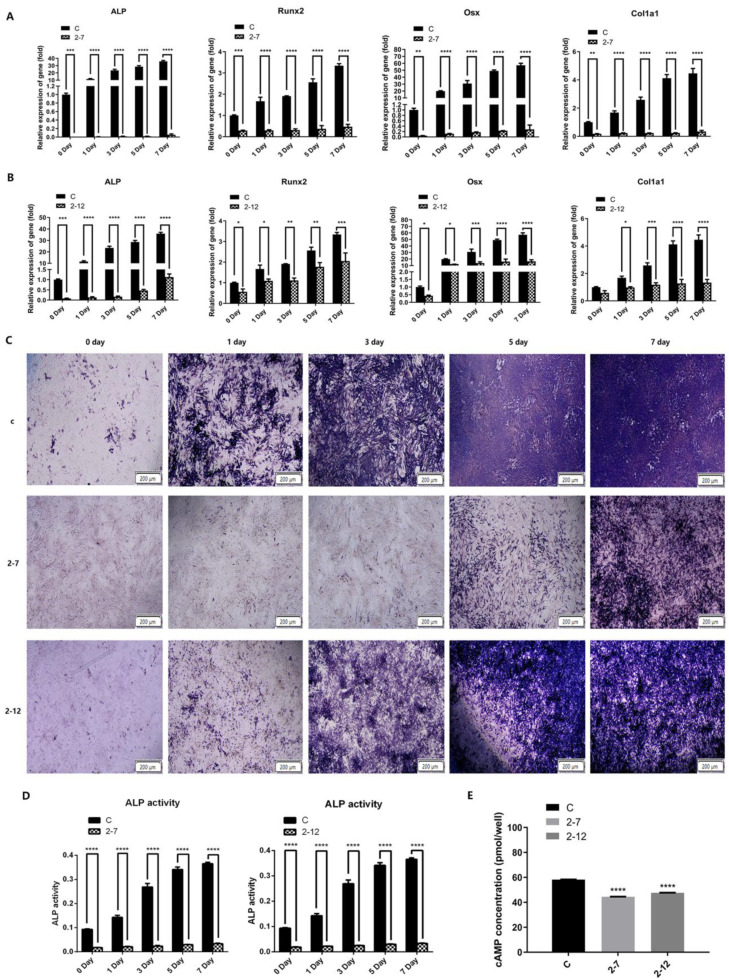
The osteogenic differentiation and the intracellular cAMP level of the NF1 cell models were reduced. (**A**) The mRNA expression levels of ALP, Runx2, Osx and Col1a1 in the 2−7 and control groups at 0, 1, 3, 5, and 7 days of osteogenic differentiation. (**B**) The mRNA expression levels of ALP, Runx2, Osx and Col1a1 in the 2−12 and control groups at 0, 1, 3, 5, and 7 days of osteogenic differentiation. (**C**) The ALP staining in the control, 2−7 and 2−12 groups at 0, 1, 3, 5, and 7 days of osteogenic differentiation. The stronger blue-purple staining represents more ALP expression. (**D**) ALP activity in the 2−7 and 2−12 groups at 0, 1, 3, 5, and 7 days of osteogenic differentiation compared to that of the control group. (**E**)The intracellular cAMP levels in the control, 2−7 and 2−12 groups. All data are presented as the mean ± SD (n = 3). * *p* < 0.05, ** *p* < 0.01, *** *p* < 0.001, **** *p* < 0.0001.

Two strains of C2C12 cells with NF1 gene knockout and the normal control group cells were all induced to undergo osteogenic differentiation. The cells were collected at 0, 1, 3, 5, and 7 days of induction to detect the expression of osteogenic differentiation marker genes, alkaline phosphatase (ALP) activity and ALP staining. Then, we investigated the effect of neurofibromin loss on C2C12 cells in terms of intracellular cAMP levels by ELISA. As shown in Figure 2A, the mRNA expression levels of ALP, Runx2, Osx and Col1a1 in the 2−7 group were significantly lower than those in the control group at different time points of osteogenic differentiation. The difference was statistically significant. As shown in Figure 2B, consistent with the 2−7 group, the mRNA expression levels of ALP, Runx2, Osx and Col1a1 in the 2−12 group were also significantly lower than those in the control group at different time points of osteogenic differentiation. The difference was statistically significant. As shown in Figure 2C, ALP staining in the control group became increasingly deeper with the prolongation of osteogenic differentiation time. However, the extent and scope of ALP staining in the two NF1 cell models were lighter and smaller than those in the control group at any time of osteogenic differentiation. The result was more pronounced in the 2−7 group. As shown in Figure 2D, both NF1 knockout cell lines 2−7 and 2−12 showed decreased ALP activity at different time points of osteogenic differentiation compared with the control cells. The trend was consistent with the expression of osteogenic differentiation marker genes and ALP staining in the two NF1 cell models. As shown in Figure 2E, after knocking out the NF1 gene, we observed the intracellular cAMP levels in 2−7 and 2−12 decreased, which was statistically significant compared with the control group. In conclusion, the above results showed that the osteogenic differentiation and the intracellular cAMP levels of the 2−7 and 2−12 strains with NF1 gene knockout were significantly lower than those of the normal control cells.

### 2.3. Icariin Improved the Osteogenic Differentiation of the NF1 Gene Knockout Cell Models at the Later Stage of the Osteogenic Induction Process

**Figure 3:**
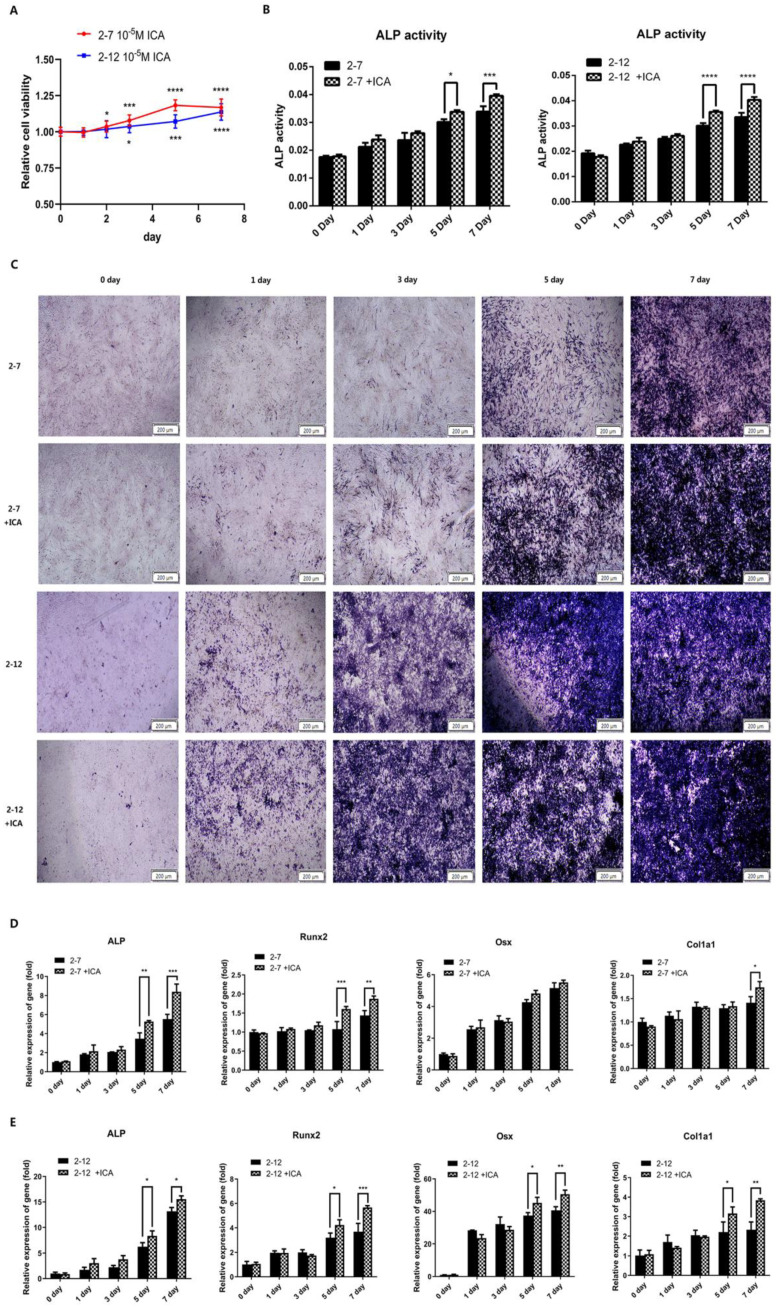
Icariin improved the osteogenic differentiation of the NF1 cell models. (**A**) The viability of the cells with icariin addition in the 2−7 or 2−12 cell lines at 0, 1, 2,3, 5, and 7 days of osteogenic differentiation. (**B**) The ALP activity in the 2−7 or 2−12 cells with icariin and the control 2−7 or 2−12 cells without icariin at 0, 1, 3, 5, and 7 days of osteogenic differentiation. (**C**) The ALP staining in the 2−7 or 2−12 cells with icariin and the control 2−7 or 2−12 cells without icariin at 0, 1, 3, 5, and 7 days of osteogenic differentiation. The stronger blue-purple staining represents more ALP expression. (**D**) The mRNA expression levels of ALP, Runx2, Osx and Col1a1 in the 2−7 group with or without icariin at 0, 1, 3, 5, and 7 days of osteogenic differentiation. (**E**) The mRNA expression levels of ALP, Runx2, Osx and Col1a1 in the 2−12 group with or without icariin at 0, 1, 3, 5, and 7 days of osteogenic differentiation. All data are presented as the mean ± SD (*n* = 3). * *p* < 0.05, ** *p* < 0.01, *** *p* < 0.001, **** *p* < 0.0001.

A previous study showed that 10^−5^ M icariin was the optimal concentration to promote the osteogenic differentiation of C2C12 cells [26]. Therefore, we used this concentration of icariin to induce osteogenic differentiation in the NF1 cell models of the subsequent experiments. The two NF1 cell lines with or without icariin treatment were collected at 0, 1, 3, 5, and 7 days of osteogenic differentiation to detect the expression of osteogenic differentiation marker genes, alkaline phosphatase (ALP) activity and ALP staining. As shown in Figure 3A, the viability of the cells gradually increased with the time of icariin addition, which was observed in both cell lines 2−7 and 2−12. The difference was statistically significant. This suggested that icariin has a proliferative effect on the NF1 knockout cells. As shown in Figure 3B, the ALP activity of the 2−7 and 2−12 groups treated with icariin was higher than those of the control group without icariin, especially at the later stage of the induction process, on the fifth and seventh days of osteogenic induction. As shown in Figure 3C, in the 2−7 group, the extent and scope of ALP staining with icariin were stronger than those without icariin treatment at 1, 3, 5, and 7 days of osteogenic differentiation. The observations in the 2−12 group were consistent with those in the 2−7 group. The extent and scope of ALP staining were also deeper and larger after the addition of icariin at 1, 3, 5, and 7 days of osteogenic differentiation. As shown in Figure 3D, the expression levels of osteogenic differentiation marker genes in the 2−7 group induced with icariin were higher than those without icariin treatment, especially at the later stage of the induction process, and the increase was the most significant at seven days of osteogenic differentiation. As shown in Figure 3E, consistent with the 2−7 group, the expression levels of osteogenic differentiation marker genes in the 2−12 group induced with icariin was higher than those without icariin treatment, and the increase was significant at both 5 and 7 days of osteogenic differentiation. The above results indicated that icariin could promote osteogenic differentiation in C2C12 cells with NF1 gene knockout.

### 2.4. RNA-Seq Analysis of Differentially Expressed Genes (DEGs) before and after Icariin Treatment during the Induction of Osteogenesis in the NF1 Cell Model Suggested cAMP Pathway Activation

**Figure 4:**
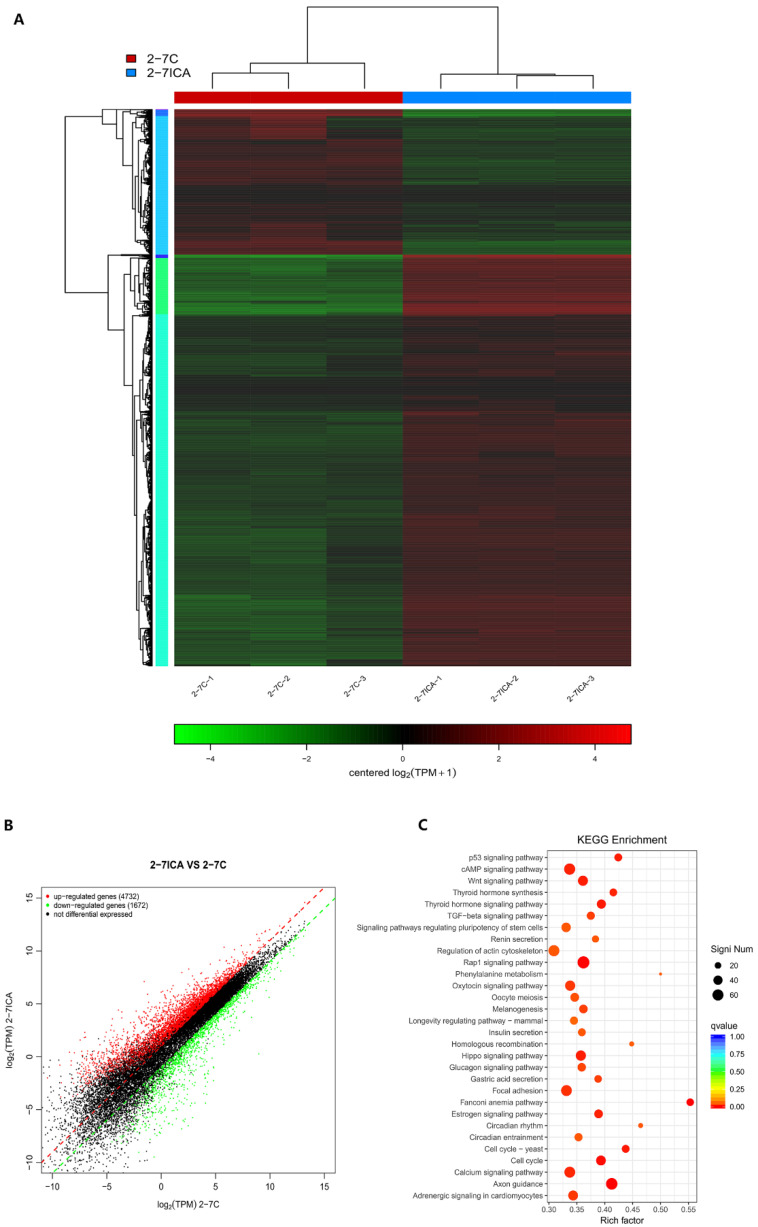
The gene expression profile of icariin-induced osteoblast differentiation in the NF1 cell model was analyzed with RNA-seq technology. (**A**) Heatmap of the differentially expressed genes in the 2−7 group treated with icariin and the 2−7 control group. Each row represents a gene, and each column represents a sample. The color indicates the level of gene expression in the sample, red indicates higher expression, and green indicates lower expression. (**B**) Scatter plot of differentially expressed genes in the comparison groups. Each point in the figure represents a gene, and the point closer to the origin has a lower expression. Red represents upregulated genes, green represents downregulated genes, and black represents nondifferentially expressed genes. Both up/down-regulation are the vertical axis samples relative to the horizontal axis samples. (**C**) Dot-plot of significantly enriched KEGG pathways. The size of the q-value is represented by the color of the dots. The smaller the q-value is, the closer the color is to the red color, and the number of differentially expressed genes contained in each pathway is represented by the size of the dots.

The differentially expressed genes were analyzed by comparing the gene expression profiles of the NF1 cell model before and after icariin treatment by transcriptome sequencing technology. The differentially expressed genes were labeled in the corresponding pathways, which can directly reflect the influence of gene expression differences in metabolic pathways and provide clues for further exploring the mechanism by which icariin promotes the osteogenic differentiation of the NF1 cell model. As shown in Figure 4A, the differentially expressed genes (DEGs) were clustered into two separate groups that coincided with the icariin-treated and untreated 2−7 group cells. Figure 4B shows that a total of 6404 DEGs, including 4732 upregulated and 1672 downregulated genes, were obtained from the comparison between icariin-treated and untreated 2−7 group cells. Next, we identified the ten most significantly upregulated and downregulated genes, and information about these genes is shown in Table 1. Information about genes is listed from top to bottom according to the significance of the differentially expressed genes. The results could help us better understand and verify the differentially expressed genes. The dot plot generated by the enrichment analysis using the KEGG database is shown in Figure 4C. KEGG analysis of DEGs in the icariin-treated 2−7 group cells compared with untreated cells suggested that the drug affected the cAMP signaling pathway.

**Figure 5:**
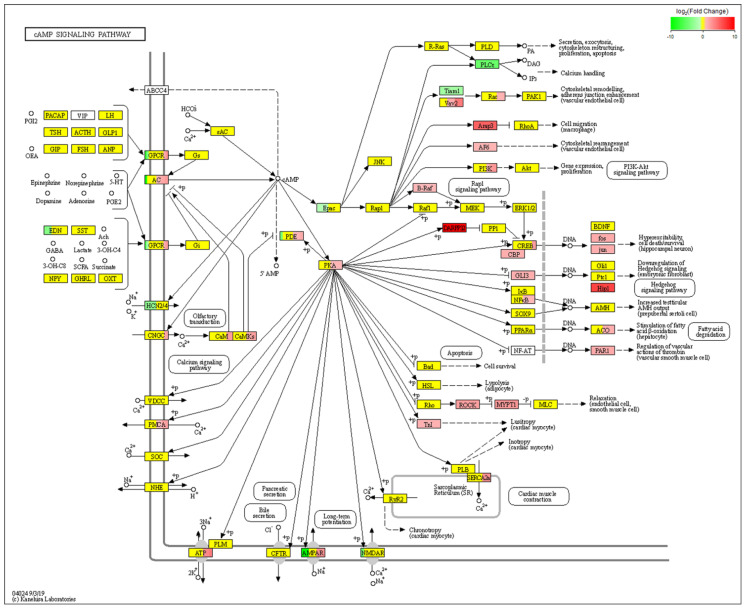
The differentially expressed genes in the cAMP metabolic pathway suggested that upregulated genes were dominant in this pathway. All the highlighted genes in the figure belong to the genes annotated in this transcriptome. The colors indicate the relative upregulation/downregulation relationship between the genes in the two comparison samples. Red indicates upregulated genes, green indicates downregulated genes, and yellow indicates nondifferentially expressed genes. The depth of the color indicates the magnitude of the upregulation or downregulation, and the darker the color, the greater the magnitude.

We next performed a KEGG pathway enrichment analysis of the 6404 DEGs and determined that a subset of DEGs was specifically enriched in the cAMP signaling pathway. The cAMP metabolic pathway in this RNA-seq analysis is shown in Figure 5 and intuitively reflects the impact of the gene expression differences in the cAMP metabolic pathway. From the cAMP signaling pathway, we found that PKA and CREB lie downstream of cAMP. We were interested in the cAMP signaling pathway because the intracellular cAMP level significantly decreased after NF1 gene knockout.

### 2.5. Icariin Activated the cAMP/PKA/CREB Pathway in the NF1 Cell Model

**Figure 6:**
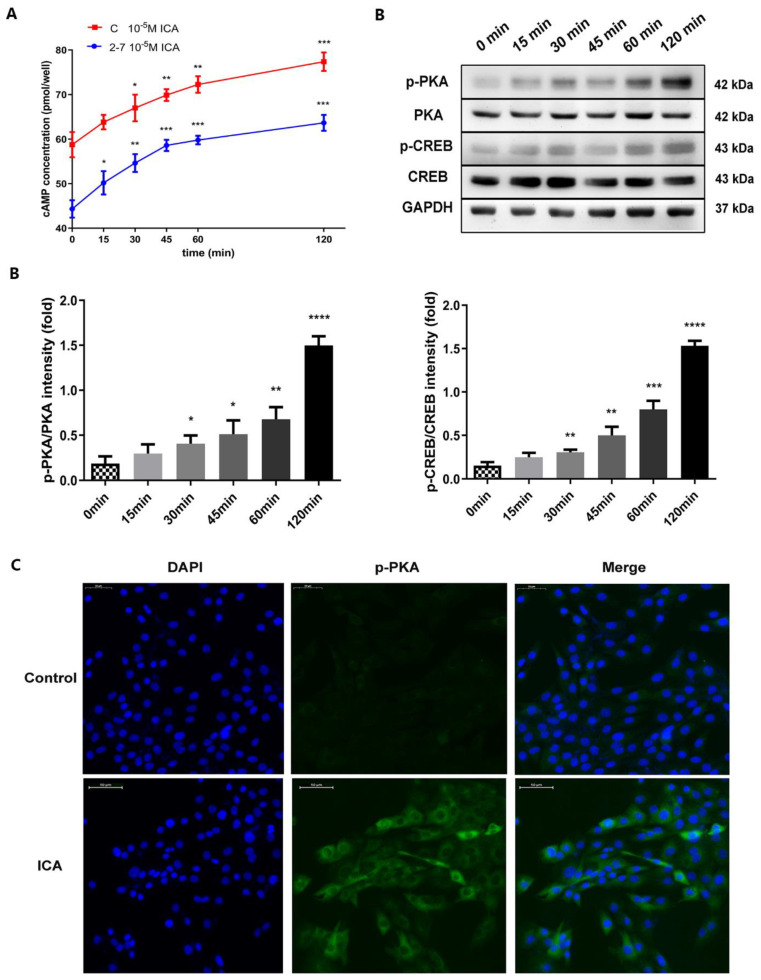
Icariin activated the cAMP/PKA/CREB pathway during osteogenic differentiation in the NF1 cell model. (**A**) Changes in intracellular cAMP levels after the 2−7 NF1 cell model and the control group exposure for 0, 15, 30, 45, 60 and 120 min. (**B**) Protein expression levels of phosphorylated PKA (p-PKA), phosphorylated CREB (p-CREB), total PKA, and CREB after the 2-7 NF1 cell model exposure for 0, 15, 30, 45, 60 and 120 min. (**C**) The expression of p-PKA after 120 min of icariin treatment by immunofluorescence assays. p-PKA was stained green, and nuclei were stained blue (with DAPI). The bar represents the mean ± SD (*n* = 3). * *p* < 0.05, ** *p* < 0.01, *** *p* < 0.001, **** *p* < 0.0001.

Our previous bioinformatics analysis provided a clue to explore further the mechanism by which icariin promotes osteogenic differentiation in the NF1 cell model. The next step was to verify changes in the expression of downstream essential proteins and target genes. After adding icariin to the osteogenic differentiation medium, we examined the changes in intracellular cAMP levels of the 2−7 cell line and control C2C12 cell at six different time points: 0, 15, 30, 45, 60 and 120 min. As shown in Figure 6A, the concentration of cAMP began to increase after 15 min in both the 2−7 cell and control cell. The increase continued with the extension of dosing time and reached the highest level at 120 min. This trend was consistent within 2−7 and the control group. The results of the Western blot are shown in Figure 6B. PKA (p-PKA) and phosphorylation of CREB (p-CREB) were significantly activated after treatment with icariin in the 2−7 NF1 cell model. With the extension of icariin treatment, we found that the expression of p-PKA and p-CREB became more pronounced, while the expression of total PKA and total CREB remained unchanged. In addition, the Western blot results were confirmed by the immunofluorescence assay of p-PKA, as shown in Figure 6C. The expression of p-PKA in the icariin 2−7 cell group was significantly higher than that in control 2−7 cell group at 120 min after icariin administration, so a more obvious fluorescence signal could be observed, while there was no visible positive expression of p-PKA in the control group. The above results showed that the increase in intracellular cAMP levels and the expression of p-PKA and p-CREB continued with the extension of icariin dosing time and reached the highest level at 120 min. Therefore, we indicated that icariin activated the cAMP/PKA/CREB pathway in the NF1 cell model.

### 2.6. A PKA Inhibitor (H89) Inhibited Icariin-Induced Osteogenesis by Blocking the cAMP/PKA/CREB Signaling Pathway in the NF1 Cell Model

**Figure 7:**
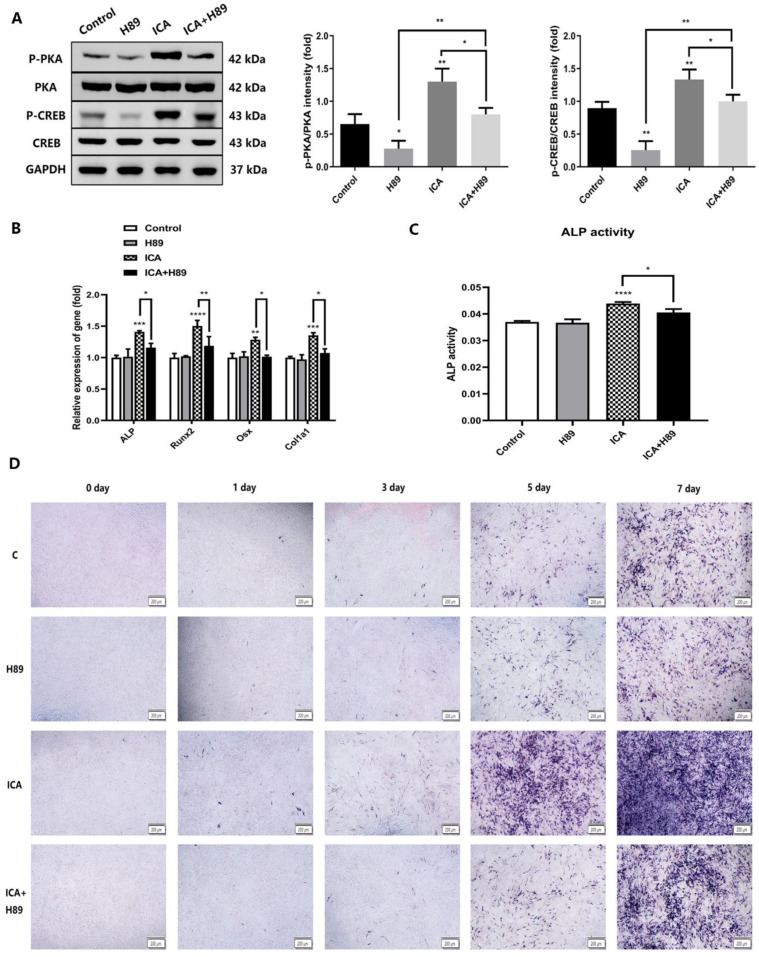
A PKA inhibitor (H89) abrogated icariin-induced osteogenesis via blockade of the cAMP/PKA/CREB signaling pathway. (**A**) The protein expression levels of p-PKA, p-CREB, total PKA and total CREB in the control group, icariin group, H89 group and icariin+H89 group. (**B**) The mRNA expression levels of ALP, Runx2, Osx and Col1a1 at seven days of osteogenic differentiation in the four cell groups. (**C**) ALP activity at seven days of osteogenic differentiation in the four cell groups. (**D**) ALP staining at 0, 1, 3, 5, and 7 days of osteogenic differentiation in the four cell groups. The deeper and larger blue-purple staining represents more ALP expression. The bar represents the mean ± SD (n = 3). * *p* < 0.05, ** *p* < 0.01, *** *p* < 0.001, **** *p* < 0.0001.

To further verify the role of cAMP signaling in the mechanism by which icariin enhances osteogenic differentiation in the NF1 cell model, we introduced a PKA inhibitor, H89, whose addition could block the activation of cAMP signaling. The 2−7 cell line was treated with osteogenic differentiation medium with or without icariin and H89 for seven days. We divided the cells into four groups: the control group, icariin group, H89 group and icariin+H89 group. As shown in Figure 7A, compared with those in the icariin group, the protein expression of p-PKA and p-CREB in the icariin+H89 group was weakened, indicating that H89 decreased the increase in p-PKA and p-CREB protein expression caused by icariin, while the expression of total PKA and total CREB did not change. Icariin-induced cAMP/PKA/CREB pathway activation was weakened by adding H89. Next, we examined the effect of H89 on the osteogenic differentiation of the NF1 cell model in terms of mRNA expression of osteogenic markers, ALP activity and ALP staining. As shown in Figure 7B, the mRNA expression levels of osteogenic differentiation marker genes in the icariin group were significantly higher than those in the control group. However, the mRNA expression level of the icariin+H89 group was significantly lower than those of the icariin group, while there was no statistically significant difference between the control group and the H89 group. The results indicated that H89 relieved the stimulatory effect of icariin on various osteogenic differentiation markers. Similarly, as shown in Figure 7C, ALP activity was significantly higher in the icariin group than in the control group. However, the increase in ALP activity in the icariin+H89 group was abolished. Consistent with the mRNA expression and ALP activity, the same trend of ALP staining is shown in Figure 7D. The extent and scope of ALP staining in the icariin group were deeper and larger than those in the icariin+H89 group. We found that the ability of icariin to enhance osteogenic differentiation in the NF1 cell model was impaired after H89 addition. In conclusion, the above results demonstrated that icariin enhanced the osteogenic differentiation of the NF1 cell model by activating the cAMP/PKA/CREB signaling pathway.

## 3. Discussion

Icariin is widely used clinically as a bone-protective agent in treating fractures and osteoporosis [27]. Studies had shown that when human bone marrow mesenchymal stem cells and primary osteoblasts of mice were cultured in vitro for osteogenic induction, the addition of icariin can promote the differentiation of primary cells into osteoblasts and promote the expression of osteoblast marker genes [28,29]. Icariin can also inhibit the differentiation of bone marrow mesenchymal stem cells into adipocytes and simultaneously increase the number of mature osteoblasts [30]. Recent studies have shown that icariin plays an essential role in promoting osteogenic differentiation of various types of osteoblasts, such as the human osteoblast line hFOB 1.19, the mouse osteoblast line MC3T3 and the mouse embryonic-derived mesenchymal stem cell line C3H10T1/2 [18,29,31]. In addition to osteogenic effects, several studies have shown that icariin inhibits the formation of various osteoclasts and prevents bone loss. Icariin suppressed osteoclastic differentiation and activity in a dose-dependent manner in the mouse osteoclast cell line RAW 264.7 [32]. Another study in postmenopausal women showed that icariin increased the bone anabolic marker and suppressed TRAF6 protein in peripheral blood osteoclast-precursor monocytes [33]. Zhang’s group found that the higher the icariin concentration was, the greater the extracellular matrix (ECM) synthesis and chondrogenic marker gene expression were [34]. Li et al. demonstrated that icariin can promote the synthesis of glycosaminoglycans and collagen in chondrocytes, which has a strong chondrogenic effect [35]. Studies have shown that icariin exhibits potent estrogenic bioactivity, antihyperglycemic effects, inhibition of glucocorticoids and stimulation of new bone formation, which can protect animal models of osteoporosis induced by ovariectomy, diabetes, glucocorticoids and OPG gene knockout [36,37,38,39]. In our research, by determining the expression of marker genes of osteogenic differentiation, such as ALP, Runx2, Osx and Col1a1, the activity and staining of alkaline phosphatase (ALP), we verified that icariin promotes osteoblast differentiation of the NF1 gene knockout cell model in vitro. We found that icariin increased the expression level of osteogenic markers and the activity or staining of ALP in a time-dependent manner. Therefore, icariin shows potential for the treatment of bone disorders in NF1. The results will provide new ideas for the clinical prevention and treatment of NF1 bone disease and drug development in the future.

RNA-Seq analysis of DEGs in our research suggested cAMP pathway activation after icariin treatment in the NF1 cell model. This phenomenon might be related to the involvement of neurofibromin in regulating adenylate cyclase activity [40]. The et al. showed that NF1−/− flies exhibited a 20 to 25% reduction in body size compared to normal flies. The fact that an exogenous increase in cAMP concentration rescued the abnormal phenotype suggested that neurofibromin can regulate the total growth of *Drosophila* through a separate RAS-independent cAMP pathway [41]. Our research found that the level of intracellular cAMP was significantly decreased in the cell models with NF1 gene knockout compared with the normal control cells, which might be the key to the induction of osteogenic disorders in NF1 cell models. Icariin was added during the induction of osteogenic differentiation, the level of intracellular cAMP was increased, the downstream pathway of cAMP was activated, and the osteogenic differentiation of the NF1 cell model was improved. Many studies have demonstrated that the cAMP/PKA signaling system, a key regulator of osteogenic lineage differentiation, regulates osteogenic differentiation and mineralization [42]. The role of the cAMP/PKA pathway in osteogenesis is supported by several studies. Parathyroid hormone (PTH) is an anabolic drug approved by the United States FDA for the treatment of osteoporosis. Many studies have shown that PTH promotes osteogenic differentiation by activating the cAMP/PKA signaling pathway [43,44]. Aditi’s group used MC3T3-E1 preosteoblasts and UMR106 rat osteosarcoma cells to demonstrate that cAMP is a second messenger delivered through the Cx43 protein and overexpression of Cx43 can significantly enhance cAMP activity and thus enhance osteoblast function [45]. Human bone marrow mesenchymal stem cells pretreated with cAMP or forskolin (an adenylate cyclase activator) showed enhanced osteogenic differentiation in vivo by activating the cAMP/PKA pathway [42,46]. Pentoxifylline is a nonselective phosphodiesterase (PDE) inhibitor that can promote osteoblast differentiation and increase bone formation by increasing cAMP levels in vitro and in vivo [47].

RNA-seq analysis in our research suggested that the DEGs not only markedly participated in cAMP signaling pathways but were also involved in Axon guidance and Rap1 signaling pathway. Enrichment of these highly related pathways may also be the mechanism of icariin in preventing and treating NF1 with skeletal defects. During the development of the nervous system, axons extend through complex environments. Ma et al. showed that mouse nerves with embryonic ectoderm development (EED) deficient Schwann cells display slow axonal regeneration with significantly decreased expression of axon guidance genes. RNA-seq analysis of EED-deficient mice identified polycomb repressive complex2-regulated molecular pathways that may contribute to the transition to malignancy in neurofibromatosis [48]. Rap1, like Ras, belongs to the GTPases of the Ras family. Both hypo- and hyperactivation of Ras/Rap signalling impair the capacity of synaptic plasticity.

Moreover, accumulating reports have linked genetic defects that either increase or decrease Ras/Rap signaling with several mental disorders associated with learning disability, such as NF1 [49]. Stornetta et al. demonstrated that NF1 gene inactivation in astrocytes results in reduced cAMP generation and attenuated calcium influx and Rap1 activation. These results showed that neurofibromin positively influences cAMP generation and activation of cAMP growth regulatory targets in astrocytes and expands the role of the NF1 gene in astrocyte growth regulation [50]. The effect on cAMP is also consistent with our results in the NF1 osteoblast model. The RNA-seq and enrichment data could be deeply analyzed, showing the biological implications of the enriched pathways possibly affected by icariin. These issues are worth exploring in the future.

In summary, we concluded that icariin could promote the osteogenic differentiation of C2C12 cells with NF1 gene knockout, and the stimulatory effect was mediated by the activation of the cAMP/PKA/CREB signaling pathway. The results of this study will provide new ideas and new targets for the prevention and treatment of NF1 bone disease and drug development from a new perspective, which has important theoretical significance and application value. However, this study also has some limitations. First, the specific connected sites and mechanisms between icariin and neurofibromin are still unclear. Next, it is necessary to construct an NF1 mouse model for further in vivo studies. For these goals, work in this direction is ongoing in our laboratory.

## 4. Materials and Methods

Reagents. Icariin reagents (purity >99%) were purchased from the National Institute for the Control of Pharmaceutical and Biological Products (Beijing, China) and dissolved in DMSO. H89 was purchased from Sigma-Aldrich (St. Louis, MO, USA). BMP2 was purchased from PeproTech (Rocky Hill, NJ, USA). Puromycin was purchased from Solarbio (Beijing, China). Antibodies against GAPDH (cat. no. 5174S), phosphorylated PKA (cat. no. 5661S), PKA (cat. no. 4782S), phosphorylated CREB (cat. no. 9198S) and CREB (cat. no. 9197T) were obtained from Cell Signaling Technology (Danvers, MA, USA). Antibody against neurofibromin (cat. no. ab17963) was purchased from Abcam (Cambridge, UK). CRISPR/Cas9 single-vector lentivirus was purchased from GeneChem (Shanghai, China).

Cell culture. The C2C12 line is a subclone of a mouse myoblast cell line. These cells were purchased from the American Type Culture Collection (ATCC) and cultured in Dulbecco’s modified Eagle’s medium (DMEM) with 10% fetal bovine serum (FBS; Gibco, CA, USA) and 1% penicillin/streptomycin in 5% CO_2_ at 37 °C. Cells were seeded; on the second day, the cells were differentiated by replacing the medium with a differentiation medium (DMEM containing 5% FBS and 300 ng/mL BMP2). Icariin was complimentary added at 10^−5^ M. The culture media were replaced every two days.

Virus transfection. When the confluence of C2C12 cells in the six-well plate reached approximately 40%, and the cells were in good condition, transfection was performed. An appropriate volume of CRISPR/Cas9 single-vector lentivirus solution with an MOI value of 200 (the highest transfection efficiency) was gently mixed into the 6-well plate. The 6-well plates were cultured in 5% CO_2_ at 37 °C.

Puromycin screening of stable cell lines. The C2C12 cells transfected with CRISPR/Cas9 single-vector lentivirus for 72 h were collected, the old medium was discarded, and the cells were washed three times with PBS. An appropriate volume of 3 µg/mL puromycin (optimal concentration) was added to the 6-well plate, mixed, and cultured in an incubator. The fresh medium was replaced every two days. After 4–5 days of screening, the negative control cells died. After ten days of screening, the transfected cells stopped dying and grew well. Next, the cells were frozen for later use.

Limited dilution method for selection of monoclonal clones. C2C12 cells were digested with trypsin to a concentration of 5 × 10^4^–10^5^ cells/mL. Then, 100 μL of growth medium (15% serum concentration) was added to each well of a 96-well plate except for well A1. Next, 200 μL of cell suspension was added to well A1, and 100 μL was removed and added to well B1 for doubling dilution. This process was performed until H1. Then, 100 μL of growth medium was added to each well from A1 to H1, and 100 μL of cell suspension was aspirated from wells A1 to H1 in column 1 and added to wells A2 to H2 in column 2 for doubling dilution. This process was repeated until column 12. Finally, the working volume was set to 200 μL. After the monoclonal cells grew, subsequent identification was performed.

Genome sequencing. Primers were designed and constructed upstream and downstream of the sgRNA target sites, and the sequences of the primers are shown in Table 2. After the genomic DNA was extracted using the QIAamp DNA Mini kit (QIAGEN, Germantown, MD, USA) according to the manufacturer’s instructions, the target gene was immediately amplified by PCR. The amplification conditions were 94 °C for 5 min, 94 °C for 30 s, and 30 cycles of 55 °C for 30 s and 72 °C for 30 s. The PCR products were analyzed by agarose gel electrophoresis to verify that the target band was in the correct position. Finally, the target band was purified and recovered for sequencing.

Alkaline phosphatase (ALP) staining. Cells were seeded in 24-well plates at a density of 2 × 10^4^ cells per well and incubated in a humidified atmosphere of 37 °C and 5% CO_2_. Cells were cultured in BMP2 with or without icariin at confluence for 1, 3, 5 and 7 days. At the end of culturing, the cells were gently washed with PBS, fixed with 4% paraformaldehyde for 10 min, and washed three times with PBS. Subsequently, the cells were permeated by a mixture of ethanol and acetone for 1 min. Finally, the cells were stained with an ALP stain kit (Wako, Japan) at 37 °C for 30 min. When necessary, excess moisture was removed from the wells, and nuclear staining was performed. The cells were observed under a microscope (EVIDENT, Tokyo, Japan) and photographed.

Alkaline phosphatase (ALP) activity assay. Cells were seeded in 24-well plates at a density of 2 × 10^4^ cells per well and incubated in a humidified atmosphere of 37 °C and 5% CO_2_. Cells were cultured in BMP2 with or without icariin at confluence for 1, 3, 5 and 7 days. At the end of culturing, cells were gently washed twice with PBS and then lysed with 0.2% Triton X-100, and the lysate was centrifuged at 14,000× *g* for 15 min. The supernatant was collected for the measurement of ALP activity by an ALP activity assay kit (Nanjing Jiancheng Bioengineering, Nanjing, China), and the protein concentrations were determined by a BCA protein assay kit (Beyotime Institute of Biotechnology, Shanghai, China).

Transcriptome sequencing. The gene expression profile of the 2-7 cell line during icariin-induced osteoblast differentiation was analyzed with RNA-seq technology. When cultures in 25 cm^2^ culture bottles reached confluence, the medium was replaced by a differentiation medium with or without icariin, and incubation was continued for seven days. The total RNA of each sample was extracted using TRIzol (Invitrogen, Albuquerque, NM, USA). The quality of the RNA was assessed by Sangon Biotech Co. (Shanghai, China) and was confirmed to pass the quality standard for building a library. Sequencing libraries were generated using VAHTSTM mRNA-seq V2 Library Prep Kit for Illumina^®^ following the manufacturer’s recommendations, and index codes were added to attribute sequences to each sample. MRNA was purified from total RNA using poly T oligo-attached magnetic beads. For the preferential selection of cDNA fragments that were 150~200 bp in length, the library fragments were purified with the AMPure XP system (Beckman Coulter, Beverly, CA, USA). Finally, PCR products were purified (AMPure XP system), and the libraries were quantified and pooled. After RNA-seq library construction, paired-end sequencing of the library was performed on HiSeq XTen sequencers (Illumina, San Diego, CA, USA). DESeq2 (version 1.12.4) was used to determine differentially expressed genes (DEGs) between two samples. Genes were considered to be significantly differentially expressed with q-value < 0.001 and |FoldChange| > 2. Gene Ontology (GO) and Kyoto Encyclopedia of Genes and Genomes (KEGG) analyses were performed to identify which DEGs were significantly enriched in GO terms or metabolic pathways. Raw and processed sequencing data files are deposited in NCBI’s SRA (PRJNA913629).

cAMP assay. When cultures in 24-well plates reached confluence, the medium was replaced by a differentiation medium with or without icariin, and incubation was continued for 0, 15, 30, 45, 60 and 120 min. At the end of culturing, cells were gently washed twice with PBS and then lysed with cell lysis buffer, and the lysate was centrifuged at 600× *g* for 10 min. Then, we determined the amounts of cAMP in wells with a cAMP enzyme immunoassay system (R&D Systems, Minneapolis, MN, USA) according to the manufacturer’s instructions.

qRT-PCR analysis. Cells were seeded in 6-well plates and treated with BMP2 (300 ng/mL or absent), ICA (10^−5^ M or absent) and H89 (10^−5^ M or absent) for 1, 3, 5 and 7 days. Total RNA was extracted using TRIzol Reagent (Invitrogen, Albuquerque, NM, USA) according to the manual. Then, the RNA samples were reverse transcribed into cDNA using a PrimeScript RT Reagent Kit with gDNA Eraser (TaKaRa, Japan). Real-time quantitative PCR detection was performed to determine the mRNA expression levels of NF1, ALP, Runx2, Osx and Col1a1 with FastStart Universal SYBR Green Master (Rox) on a LightCycler^®^ 480 II Real-Time PCR System (Roche, Mannheim, Germany). The conditions were 95 °C for 5 min, 95 °C for 30 s, and 45 cycles of 60 °C for 30 s and 72 °C for 45 s. All reactions were run in triplicate, and data were analyzed using the 2^−ΔΔCt^ method normalized to GAPDH. The primer sequences are listed in Table 3.

Western blot analysis. Cells seeded in 25 cm^2^ culture bottles were lysed with cold RIPA Lysate reagent (Beyotime Institute of Biotechnology, Shanghai, China) according to the manufacturer’s instructions. The protein concentrations were measured with the BCA assay kit. Proteins were separated by 12% SDS-PAGE and transferred to 0.45 µm PVDF membranes (Merck Millipore, Darmstadt, Hesse-Darmstadt, Germany). After the membranes were blocked, they were incubated overnight at 4 °C with the diluted primary antibodies against the following molecules: neurofibromin, PKA, phosphorylated PKA, CREB, phosphorylated CREB and GAPDH. Then, the membranes were washed with TBST and incubated with diluted secondary antibodies for 1 h at room temperature. Finally, we visualized the membranes with an enhanced ECL substrate kit (Millipore, Billerica, MA, USA) on the Fusion SOLO S (Vilber, Collegien, France).

*Immunofluorescence staining.* Cells were seeded on coverslips in 24-well plates, fixed with 4% paraformaldehyde for 30 min, and washed thrice with PBS. Then, the cells were permeabilized with 0.5% Triton X-100 for 20 min. Subsequently, the cells were blocked in 5% goat serum for 1 h at room temperature and then incubated with an anti-phosphorylated PKA antibody overnight at 4 °C. The next day, we rewarmed the cells at room temperature for 1 h and then incubated the cells with a goat anti-rabbit conjugated secondary antibody for 1 h at 37 °C in the dark. Finally, the cells were incubated with DAPI for 5 min. The slides were examined using a 3D scanner (3D HISTECH, Budapest, Hungary).

*Statistical analysis.* All data are expressed as the mean ± SD. Each treatment group had at least three replicates (n = 3), and each experiment was repeated three times. Statistical analyses were conducted with PRISM (version 8, GraphPad Software, Inc., San Diego, CA, USA). The differences between two groups and multiple groups were evaluated using an unpaired *t*-test and a one-way analysis of variance, respectively. In all cases, *p* < 0.05 was considered significant.

## Figures and Tables

**Table 1:** The ten most significantly upregulated and downregulated genes.

Gene Id	Gene Name	MeanTPM (2−7 ICA)	MeanTPM (2−7 C)	log2FoldChange	*p* Value	q Value	Result
ENSMUSG00000101859	*Gm29233*	7.683309	0.0001	16.22944016	4.74 × 10^−9^	2.18 × 10^−8^	up
ENSMUSG00000023078	*Cxcl13*	7.085217333	0.0001	16.11252449	1.21 × 10^−14^	8.37 × 10^−14^	up
ENSMUSG00000055874	*Foxi3*	6.177744333	0.0001	15.91479255	1.27 × 10^−14^	8.73 × 10^14^	up
ENSMUSG00000048368	*Omd*	6.120898667	0.0001	15.90145586	5.23 × 10^−16^	3.92 × 10^−15^	up
ENSMUSG00000036960	*Clca2*	5.547391	0.0001	15.75952179	5.21 × 10^−18^	4.34 × 10^−17^	up
ENSMUSG00000084932	*Gm15156*	4.823441667	0.0001	15.5577753	1.21 × 10^−8^	5.36 × 10^−08^	up
ENSMUSG00000077285	*Gm26394*	3.750423	0.0001	15.1947657	0.00138128	0.003356227	up
ENSMUSG00000098698	*Mir6960*	3.651845	0.0001	15.15633791	0.02583538	0.048832188	up
ENSMUSG00000062773	*Tex101*	3.636343	0.0001	15.15020067	1.55 × 10^−11^	8.75 × 10^−11^	up
ENSMUSG00000041552	*Ptchd1*	3.509552667	0.0001	15.09899953	1.68 × 10^−14^	1.15 × 10^−13^	up
ENSMUSG00000000730	*Dnmt3l*	0.0001	11.46687133	−16.80711229	8.03 × 10^−18^	6.61 × 10^−17^	down
ENSMUSG00000078302	*Foxd1*	0.0001	4.958846333	−15.5977169	1.45 × 10^−18^	1.25 × 10^−17^	down
ENSMUSG00000036938	*Try5*	0.0001	3.806281333	−15.21609458	2.33 × 10^−12^	1.38 × 10^−11^	down
ENSMUSG00000112148	*Lilrb4a*	0.0001	3.492889	−15.09213318	4.60 × 10^−15^	3.24 × 10^−14^	down
ENSMUSG00000000732	*Icosl*	0.0001	3.242253	−14.98470905	5.86 × 10^−17^	4.64 × 10^−16^	down
ENSMUSG00000035513	*Ntng2*	0.0001	3.095218333	−14.91775356	3.13 × 10^−14^	2.10 × 10^−13^	down
ENSMUSG00000021464	*Ror2*	0.0001	2.735676667	−14.73961011	1.46 × 10^−18^	1.26 × 10^−17^	down
ENSMUSG00000031548	*Sfrp1*	0.0001	2.401819667	−14.55184021	3.84 × 10^−18^	3.22 × 10^−17^	down
ENSMUSG00000050578	*Mmp13*	0.0001	2.337899333	−14.51292519	1.01 × 10^−15^	7.45 × 10^−15^	down
ENSMUSG00000040867	*Begain*	0.0001	2.319114333	−14.50128633	3.46 × 10^−15^	2.46 × 10^−14^	down

**Table 2:** Primers used for PCR.

Name	Sequence
*NF1*	3′→5′: GGGTCGGGCTTCAATGGTAA 5′→3′: TCCCACATGCTTTAGGCACT

**Table 3:** Primers used for qRT-PCR.

Name	Sequence
*NF1*	3′→5′: TTCGGATAAGCCCTCACAACAAC 5′→3′: CAGCATCAATCTTAGGCCACCA
*Alp*	3′→5′: GGCTCTGCCTTTATTCCCTAGT 5′→3′: AAATAAGGTGCTTTGGGAATCTGT
*Runx2*	3′→5′: GCCGGGAATGATGAGAACTA 5′→3′: GGTGAAACTCTTGCCTCGTC
*Osx*	3′→5′: AGGCCTTTGCCAGTGCCTA 5′→3′:GCCAGATGGAAGCTGTGAAGA
*Col1a1*	3′→5′: GACATGTTCAGCTTTGTGGACCTC 5′→3′:GGGACCCTTAGGCCATTGTGTA
*Gapdh*	3′→5′: CATCCCAGAGCTGAACG 5′→3′: CTGGTCCTCAGTGTAGCC

## Data Availability

The datasets generated and analyzed during the present study are available from the corresponding author upon reasonable request.

## Sample Availability

Not available.

## References

[1] Ge L.-L., Xing M.-Y., Zhang H.-B., Wang Z.-C. (2022). Neurofibroma Development in Neurofibromatosis Type 1: Insights from Cellular Origin and Schwann Cell Lineage Development. Cancers.

[2] Elefteriou F., Kolanczyk M., Schindeler A., Viskochil D.H., Hock J.M., Schorry E.K., Crawford A.H., Friedman J.M., Little D., Peltonen J. (2009). Skeletal abnormalities in neurofibromatosis type 1: Approaches to therapeutic options. Am. J. Med. Genet. A.

[3] de la Croix Ndong J., Stevens D.M., Vignaux G., Uppuganti S., Perrien D.S., Yang X., Nyman J.S., Harth E., Elefteriou F. (2015). Combined MEK inhibition and BMP2 treatment promotes osteoblast differentiation and bone healing in Nf1Osx -/- mice. J. Bone Miner. Res..

[4] Kaspiris A., Savvidou O.D., Vasiliadis E.S., Hadjimichael A.C., Melissaridou D., Iliopoulou-Kosmadaki S., Iliopoulos I.D., Papadimitriou E., Chronopoulos E. (2022). Current Aspects on the Pathophysiology of Bone Metabolic Defects during Progression of Scoliosis in Neurofibromatosis Type 1. J. Clin. Med..

[5] Kolanczyk M., Kossler N., Kühnisch J., Lavitas L., Stricker S., Wilkening U., Manjubala I., Fratzl P., Spörle R., Herrmann B.G. (2007). Multiple roles for neurofibromin in skeletal development and growth. Hum. Mol. Genet..

[6] Moramarco A., Mallone F., Sacchetti M., Lucchino L., Miraglia E., Roberti V., Lambiase A., Giustini S. (2021). Hyperpigmented spots at fundus examination: A new ocular sign in Neurofibromatosis Type I. Orphanet. J. Rare Dis..

[7] Ratner N., Miller S.J. (2015). A RASopathy gene commonly mutated in cancer: The neurofibromatosis type 1 tumour suppressor. Nat. Rev. Cancer.

[8] Machado Almeida P., Lago Solis B., Stickley L., Feidler A., Nagoshi E. (2021). Neurofibromin 1 in mushroom body neurons mediates circadian wake drive through activating cAMP-PKA signaling. Nat. Commun..

[9] Abramowicz A., Gos M. (2014). Neurofibromin in neurofibromatosis type 1—Mutations in NF1gene as a cause of disease. Dev. Period Med..

[10] Petramala L., Giustini S., Zinnamosca L., Marinelli C., Colangelo L., Cilenti G., Formicuccia M.C., D’Erasmo E., Calvieri S., Letizia C. (2012). Bone mineral metabolism in patients with neurofibromatosis type 1 (von Recklingausen disease). Arch Dermatol. Res..

[11] Fowlkes J.L., Thrailkill K.M., Bunn R.C. (2021). RASopathies: The musculoskeletal consequences and their etiology and pathogenesis. Bone.

[12] Tucker T., Schnabel C., Hartmann M., Friedrich R.E., Frieling I., Kruse H.P., Mautner V.F., Friedman J.M. (2009). Bone health and fracture rate in individuals with neurofibromatosis 1 (NF1). J. Med. Genet..

[13] Stevenson D.A., Schwarz E.L., Viskochil D.H., Moyer-Mileur L.J., Murray M., Firth S.D., D’Astous J.L., Carey J.C., Pasquali M. (2008). Evidence of increased bone resorption in neurofibromatosis type 1 using urinary pyridinium crosslink analysis. Pediatr. Res..

[14] Lee D.Y., Cho T.-J., Lee H.R., Lee K., Moon H.J., Park M.S., Yoo W.J., Chung C.Y., Choi I.H. (2011). Disturbed osteoblastic differentiation of fibrous hamartoma cell from congenital pseudarthrosis of the tibia associated with neurofibromatosis type I. Clin. Orthop. Surg..

[15] Kuorilehto T., Nissinen M., Koivunen J., Benson M.D., Peltonen J. (2004). NF1 tumor suppressor protein and mRNA in skeletal tissues of developing and adult normal mouse and NF1-deficient embryos. J. Bone Miner. Res..

[16] Wu X., Estwick S.A., Chen S., Yu M., Ming W., Nebesio T.D., Li Y., Yuan J., Kapur R., Ingram D. (2006). Neurofibromin plays a critical role in modulating osteoblast differentiation of mesenchymal stem/progenitor cells. Hum. Mol. Genet..

[17] Elefteriou F., Benson M.D., Sowa H., Starbuck M., Liu X., Ron D., Parada L.F., Karsenty G. (2006). ATF4 mediation of NF1 functions in osteoblast reveals a nutritional basis for congenital skeletal dysplasiae. Cell Metab..

[18] Liang W., Lin M., Li X., Li C., Gao B., Gan H., Yang Z., Lin X., Liao L., Yang M. (2012). Icariin promotes bone formation via the BMP-2/Smad4 signal transduction pathway in the hFOB 1.19 human osteoblastic cell line. Int. J. Mol. Med..

[19] Zhang D., Zhang J., Fong C., Yao X., Yang M. (2012). Herba epimedii flavonoids suppress osteoclastic differentiation and bone resorption by inducing G2/M arrest and apoptosis. Biochimie.

[20] Shi W., Gao Y., Wang Y., Zhou J., Wei Z., Ma X., Ma H., Xian C.J., Wang J., Chen K. (2017). The flavonol glycoside icariin promotes bone formation in growing rats by activating the cAMP signaling pathway in primary cilia of osteoblasts. J. Biol. Chem..

[21] Kuorilehto T., Pöyhönen M., Bloigu R., Heikkinen J., Väänänen K., Peltonen J. (2005). Decreased bone mineral density and content in neurofibromatosis type 1: Lowest local values are located in the load-carrying parts of the body. Osteoporos. Int..

[22] Yaffe D., Saxel O. (1977). Serial passaging and differentiation of myogenic cells isolated from dystrophic mouse muscle. Nature.

[23] Won G.W., Sung M., Lee Y., Lee Y.H. (2019). MST2 kinase regulates osteoblast differentiation by phosphorylating and inhibiting Runx2 in C2C12 cells. Biochem. Biophys. Res. Commun..

[24] Soundharrajan I., Kim D.H., Srisesharam S., Kuppusamy P., Sivanesan R., Choi K.C. (2018). Limonene promotes osteoblast differentiation and 2-deoxy-d-glucose uptake through p38MAPK and Akt signaling pathways in C2C12 skeletal muscle cells. Phytomedicine.

[25] Hidaka Y., Chiba-Ohkuma R., Karakida T., Onuma K., Yamamoto R., Fujii-Abe K., Saito M.M., Yamakoshi Y., Kawahara H. (2020). Combined Effect of Midazolam and Bone Morphogenetic Protein-2 for Differentiation Induction from C2C12 Myoblast Cells to Osteoblasts. Pharmaceutics.

[26] Chen M., Cui Y., Li H., Luan J., Zhou X., Han J. (2019). Icariin Promotes the Osteogenic Action of BMP2 by Activating the cAMP Signaling Pathway. Molecules.

[27] Fan J.-J., Cao L.-G., Wu T., Wang D.-X., Jin D., Jiang S., Zhang Z.-Y., Bi L., Pei G.-X. (2011). The dose-effect of icariin on the proliferation and osteogenic differentiation of human bone mesenchymal stem cells. Molecules.

[28] Luo G., Xu B., Wang W., Wu Y., Li M. (2018). Study of the osteogenesis effect of icariside II and icaritin on canine bone marrow mesenchymal stem cells. J. Bone Miner. Metab..

[29] Zhao J., Ohba S., Shinkai M., Chung U.-I., Nagamune T. (2008). Icariin induces osteogenic differentiation in vitro in a BMP- and Runx2-dependent manner. Biochem. Biophys. Res. Commun..

[30] Zhang D., Fong C., Jia Z., Cui L., Yao X., Yang M. (2016). Icariin Stimulates Differentiation and Suppresses Adipocytic Transdifferentiation of Primary Osteoblasts Through Estrogen Receptor-Mediated Pathway. Calcif. Tissue Int..

[31] Wu Z., Ou L., Wang C., Yang L., Wang P., Liu H., Xiong Y., Sun K., Zhang R., Zhu X. (2017). Icaritin induces MC3T3-E1 subclone14 cell differentiation through estrogen receptor-mediated ERK1/2 and p38 signaling activation. Biomed. Pharmacother..

[32] Liu Y.-Q., Yang Q.-X., Cheng M.-C., Xiao H.-B. (2014). Synergistic inhibitory effect of Icariside II with Icaritin from Herba Epimedii on pre-osteoclastic RAW264.7 cell growth. Phytomedicine.

[33] Yong E.-L., Cheong W.F., Huang Z., Thu W.P.P., Cazenave-Gassiot A., Seng K.Y., Logan S. (2021). Randomized, double-blind, placebo-controlled trial to examine the safety, pharmacokinetics and effects of Epimedium prenylflavonoids, on bone specific alkaline phosphatase and the osteoclast adaptor protein TRAF6 in post-menopausal women. Phytomedicine.

[34] Zhang L., Zhang X., Li K.-F., Li D.-X., Xiao Y.-M., Fan Y.-J., Zhang X.-D. (2012). Icariin promotes extracellular matrix synthesis and gene expression of chondrocytes in vitro. Phytother. Res..

[35] Li D., Yuan T., Zhang X., Xiao Y., Wang R., Fan Y., Zhang X. (2012). Icariin: A potential promoting compound for cartilage tissue engineering. Osteoarthr. Cartil..

[36] Qi S., He J., Zheng H., Chen C., Lan S. (2019). Icariin Prevents Diabetes-Induced Bone Loss in Rats by Reducing Blood Glucose and Suppressing Bone Turnover. Molecules.

[37] Li G.-W., Xu Z., Chang S.-X., Nian H., Wang X.-Y., Qin L.-D. (2014). Icariin prevents ovariectomy-induced bone loss and lowers marrow adipogenesis. Menopause.

[38] Feng R., Feng L., Yuan Z., Wang D., Wang F., Tan B., Han S., Li T., Li D., Han Y. (2013). Icariin protects against glucocorticoid-induced osteoporosis in vitro and prevents glucocorticoid-induced osteocyte apoptosis in vivo. Cell Biochem. Biophys..

[39] Li X.-F., Xu H., Zhao Y.-J., Tang D.-Z., Xu G.-H., Holz J., Wang J., Cheng S.-D., Shi Q., Wang Y.-J. (2013). Icariin Augments Bone Formation and Reverses the Phenotypes of Osteoprotegerin-Deficient Mice through the Activation of Wnt/β-Catenin-BMP Signaling. Evid. Based Complement Alternat. Med..

[40] Izawa I., Tamaki N., Saya H. (1996). Phosphorylation of neurofibromatosis type 1 gene product (neurofibromin) by cAMP-dependent protein kinase. FEBS Lett..

[41] The I., Hannigan G.E., Cowley G.S., Reginald S., Zhong Y., Gusella J.F., Hariharan I.K., Bernards A. (1997). Rescue of a Drosophila NF1 mutant phenotype by protein kinase A. Science.

[42] Siddappa R., Martens A., Doorn J., Leusink A., Olivo C., Licht R., van Rijn L., Gaspar C., Fodde R., Janssen F. (2008). cAMP/PKA pathway activation in human mesenchymal stem cells in vitro results in robust bone formation in vivo. Proc. Natl. Acad. Sci. USA.

[43] Chen B., Lin T., Yang X., Li Y., Xie D., Cui H. (2016). Intermittent parathyroid hormone (1-34) application regulates cAMP-response element binding protein activity to promote the proliferation and osteogenic differentiation of bone mesenchymal stromal cells, via the cAMP/PKA signaling pathway. Exp. Ther. Med..

[44] Nakao Y., Koike T., Ohta Y., Manaka T., Imai Y., Takaoka K. (2009). Parathyroid hormone enhances bone morphogenetic protein activity by increasing intracellular 3′,5′-cyclic adenosine monophosphate accumulation in osteoblastic MC3T3-E1 cells. Bone.

[45] Gupta A., Anderson H., Buo A.M., Moorer M.C., Ren M., Stains J.P. (2016). Communication of cAMP by connexin43 gap junctions regulates osteoblast signaling and gene expression. Cell Signal.

[46] Doorn J., Siddappa R., van Blitterswijk C.A., de Boer J. (2012). Forskolin enhances in vivo bone formation by human mesenchymal stromal cells. Tissue Eng. Part A.

[47] Tsutsumimoto T., Wakabayashi S., Kinoshita T., Horiuchi H., Takaoka K. (2002). A phosphodiesterase inhibitor, pentoxifylline, enhances the bone morphogenetic protein-4 (BMP-4)-dependent differentiation of osteoprogenitor cells. Bone.

[48] Ma K.H., Duong P., Moran J.J., Junaidi N., Svaren J. (2018). Polycomb repression regulates Schwann cell proliferation and axon regeneration after nerve injury. Glia.

[49] Stornetta R.L., Zhu J.J. (2011). Ras and Rap signaling in synaptic plasticity and mental disorders. Neuroscientist.

[50] Dasgupta B., Dugan L.L., Gutmann D.H. (2003). The neurofibromatosis 1 gene product neurofibromin regulates pituitary adenylate cyclase-activating polypeptide-mediated signaling in astrocytes. J. Neurosci..

